# High-Quality Draft Genome Sequences of Rare Nontuberculous Mycobacteria Isolated from Surfaces of a Hospital

**DOI:** 10.1128/MRA.00496-19

**Published:** 2019-05-23

**Authors:** Igor Tiago, Susana Alarico, Ana Maranha, Catarina Coelho, Sónia Gonçalves Pereira, Nuno Empadinhas

**Affiliations:** a Centre for Functional Ecology (CFE), Department of Life Sciences, University of Coimbra, Coimbra, Portugal; b Center for Neuroscience and Cell Biology (CNC), University of Coimbra, Coimbra, Portugal; c Institute for Interdisciplinary Research (IIIUC), University of Coimbra, Coimbra, Portugal; University of Delaware

## Abstract

Nontuberculous mycobacteria (NTM), some of which had multidrug-resistant profiles, were isolated from a tertiary care hospital setting. Although most NTM are nonpathogenic, contamination of hospital surfaces by these opportunistic pathogens poses a health risk to vulnerable inpatients. These high-quality NTM draft genomes are fundamental for future genetic and epidemiological studies.

## Abstract (precis)

Nontuberculous mycobacteria (NTM), some of which had multidrug-resistant profiles, were isolated from a tertiary care hospital setting. Although most NTM are nonpathogenic, contamination of hospital surfaces by these opportunistic pathogens poses a health risk to vulnerable inpatients.

## ANNOUNCEMENT

Nontuberculous mycobacteria (NTM) are ubiquitous in nature and in human-engineered environments. Their classification and clinical relevance have been considered since the 1950s, owing to the efforts of Ernest Runyon, in understanding the role of these organisms in atypical human infections acquired from the environment (1, 2). Despite their opportunism and high resistance to standard therapeutic antibiotics and disinfectants, which have been widely acknowledged, their prevalence in anthropogenic environments and real impact on human health are still neglected (3).

Recent phylogenomic analyses reclassified the genus *Mycobacterium* into five distinct genera, namely, *Mycobacterium*, *Mycolicibacterium*, *Mycolicibacter*, *Mycolicibacillus*, and *Mycobacteroides* (4, 5).

We have performed a microbiological survey aimed at the investigation of the presence of NTM populations in a tertiary care hospital (6). Here, we present the high-quality draft genome sequences of the five NTM strains isolated from surfaces of different wards in that hospital. Samples were recovered using swabs to sample each surface and transported in tubes containing peptone water and after 3 h of shaking were used to inoculate Middlebrook 7H10-PANTA medium (PANTA medium contains an antibiotic mixture of polymyxin B, amphotericin B, nalidixic acid, trimethoprim, and azlocillin) supplemented with 10% oleic acid-albumin-dextrose-catalase (OADC) (6). The phylogenetic classification of the isolates was performed by concatenation of partial sequences of the 16S rRNA, *rpoB*, and *hsp65* genes. Isolates 10AIII, 29AIII, and 35AIII were classified as closely related to strains of Mycobacterium paragordonae, while isolate 22DIII was considered to be related to strains of Mycolicibacterium obuense and isolate 24AIII to strains of Mycolicibacterium mucogenicum. Remarkably, the three *M. paragordonae* strains displayed differences in their antibiotic susceptibility profiles, while the *M. obuense* and M. mucogenicum strains were found to be resistant to several CLSI-recommended drugs (6).

The release of the draft genome sequences of these NTM strains recovered from small areas of hospital surfaces is indicative of a potentially significant ward contamination and is relevant for future population epidemiologic and genetic studies since they pose a potential threat to vulnerable inpatients.

The genomic DNA of the five NTM pure cultures grown in the medium used for isolation as described above was extracted using a protocol adapted from Nielsen et al., with initial incubation for 2 h at 37°C in glucose Tris-EDTA (GTE) buffer (50 mM glucose, 25 mM Tris-HCl at pH 8.0, and 10 mM EDTA) containing lysozyme (20 mg/ml) (7, 8). Libraries were prepared using the Nextera XT library prep workflow (Illumina), and 2 × 150-nucleotide (nt) paired-end reads were generated on an Illumina MiSeq instrument. Quality trimming was executed using the sliding-window operation in TrimGalore (9) with default parameters. The final assembly was performed using the SPAdes (10) assembler (version 3.50) using kmers of 33, 55, and 77 nt. The assembly was subjected to binning with MetaBAT (11), and a quality check was performed on the final resulting file with CheckM (12). The high-quality-draft genome sequences were used to determine DNA-DNA hybridization (DDH) values (13) against the type strain genomes presented at NCBI GenBank and corroborate the phylogenetic classification described above. DDH values, metadata, and *de novo* assembly values are shown in Table 1.

**TABLE 1:** Data regarding phylogenetic assignment, raw data, and *de novo* assembly results for the five strains in this study

Strain	Species	No. of high-quality raw sequences	No. of contigs	*N*_50_ (bp)	Draft genome size (bp)	No. of coding sequences	G+C content (%)	Coverage (×)	DDH (%)*^a^*	Model CI (%)^a^	GenBank accession no.
10AIII	Mycobacterium paragordonae	37,870,761	60	210,802	7,046,008	6,372	66.9	331	83.4	80.5–85.8	SDLQ00000000
29AIII	Mycobacterium paragordonae	38,831,078	49	1,195,780	6,697,711	6,015	67.1	308	83.7	80.9–86.2	SDLN00000000
35AIII	Mycobacterium paragordonae	44,929,625	48	286,333	7,049,864	6,335	66.9	317	84.6	81.8–87	SDLM00000000
22DIII	*Mycolicibacterium obuense*	9,985,256	16	467,358	5,599,206	5,314	68	92	90.5	87.3–92.9	SDLP00000000
24AIII	*Mycolicibacterium mucogenicum*	10,783,531	47	239,262	5,494,553	5,331	67.4	113	72.1	69.1–74.9	SDLO00000000

a Values presented refer to those calculated by formula 2 as recommended at https://ggdc.dsmz.de, where the calculations were made. CI, confidence interval.

The assembled genomes were annotated using the NCBI Prokaryotic Genomes Annotation Pipeline (PGAP) and have been deposited at DDBJ/EMBL/GenBank.

### Data availability.

These whole-genome shotgun projects have been deposited at DDBJ/ENA/GenBank under the accession numbers SDLQ00000000, SDLP00000000, SDLO00000000, SDLN00000000, and SDLM00000000. Strains are available from the authors upon request. Raw sequencing reads for the strains are available in the NCBI Sequence Read Archive under accession numbers SRR8483011 to SRR8483015.
